# Evaluating the implementation of the community mental health program GBV: service users’ perspectives

**DOI:** 10.1192/j.eurpsy.2025.1490

**Published:** 2025-08-26

**Authors:** A. S. Mueller-Stierlin, S. Reuter, R. Kilian

**Affiliations:** 1 Institute for Epidemiology and Medical Biometry; 2 Department of Psychiatry II, University of Ulm, Ulm, Germany

## Abstract

**Introduction:**

A multi-site randomized controlled study demonstrated that the community mental health intervention GBV, when combined with standard care in the German healthcare system, led to greater improvements in empowerment, quality of life, and needs-orientation for people living with severe mental illness. However, to gain a comprehensive understanding, it is essential to include service users’ perspectives alongside effectiveness data.

**Objectives:**

This study aimed to assess the implementation of GBV from the service users’ viewpoint, providing a holistic evaluation of the intervention beyond randomized trial results.

**Methods:**

A mixed-methods approach was used to evaluate the service users’ experiences with GBV. Semi-structured interviews were conducted, transcribed, and analysed using thematic content analysis. Additionally, fidelity ratings were collected after 12 months of intervention, based on a scale developed from GBV quality standards, focusing on needs orientation, relationship building, and service availability.

**Results:**

The process evaluation revealed a greater increase in empowerment, subjective quality of life and treatment satisfaction as well as a greater reduction in unmet needs with a subjectively higher perceived manual fidelity. Qualitative interviews supported these findings, emphasizing the critical role of strong relationships with GBV staff, personalized care tailored to individual needs, and adherence to GBV’s quality criteria.

**Conclusions:**

While the effectiveness of GBV has been established, ensuring fidelity to the intervention’s manual is crucial for large-scale implementation. Key factors for success include a focus on relationship building and needs-orientation, ensuring that service delivery aligns with the predefined GBV quality standards.

**Disclosure of Interest:**

None Declared

